# Yeasts producing zeatin

**DOI:** 10.7717/peerj.6474

**Published:** 2019-02-20

**Authors:** Rostislav A. Streletskii, Aleksey V. Kachalkin, Anna M. Glushakova, Andrey M. Yurkov, Vladimir V. Demin

**Affiliations:** 1 Soil Science Faculty, Lomonosov Moscow State University, Moscow, Russia; 2 All-Russian Collection of Microorganisms (VKM), G.K.Skryabin Institute of Biochemistry and Physiology of Microorganisms RAS, Pushchino, Russia; 3 DSMZ-German Collection of Microorganisms and Cell Cultures, Leibniz Institute, Braunschweig, Germany

**Keywords:** Phytohormones, Yeast, Zeatin, HPLC-MS/MS, Cytokinins

## Abstract

The present paper describes the first screening study of the ability of natural yeast strains to synthesize in culture the plant-related cytokine hormone zeatin, which was carried out using HPLC-MS/MS. A collection of 76 wild strains of 36 yeast species (23 genera) isolated from a variety of natural substrates was tested for the production of zeatin using HPLC-MS/MS. Zeatin was detected in more than a half (55%) of studied strains and was more frequently observed among basidiomycetous than ascomycetous species. The amount of zeatin accumulated during the experiment varied among species and strains. Highest zeatin values were recorded for basidiomycete *Sporobolomyces roseus* and ascomycete *Taphrina* sp. that produced up to 8,850.0 ng and 5,166.4 ng of zeatin per g of dry biomass, respectively. On average, the ability to produce zeatin was more pronounced among species isolated from the arctic-alpine zone than among strains from tropical and temperate climates. Our study also demonstrated that epiphytic strains and pigmented yeast species, typically for phyllosphere, are able to more often produce a plant hormone zeatin than other yeasts.

## Introduction

Growth and development of plant stems, roots and fruits are largely controlled by plant growth promoters (plant hormones or phytohormones) such as abscisic acid, gibberellins, auxins and cytokinins. Cytokinins are organic molecules that promote plant growth through facilitated cellular division and growth. Zeatin is one of the most common cytokinins and, like other adenine-derived cytokinins, is synthesized in plant roots (Li, Li & Smith, 2017). Although plant hormones are naturally synthesized in plants, similar compounds are produced by bacteria and fungi. In particular, cytokinins produced by microorganisms have been suggested to promote the growth in higher plants (Arshad & Frankenberger, 1991; Tsavkelova et al., 2006; Arkhipova et al., 2007; Duca et al., 2014; Kudoyarova et al., 2014). Research on yeasts producing plant growth promoters in culture has been intensified in recent years. Several species were reported to synthesize auxins, mainly indole-3-acetic acid or IAA (Limtong & Koowadjanakul, 2012; Limtong et al., 2014; Nutaratat et al., 2014; Ignatova et al., 2015; Jaiboon et al., 2016; Streletskii et al., 2016). Though synthesis of auxins is rather widespread among yeasts (e.g. Streletskii et al., 2016, Kemler et al., 2017), studies focused on other plant growth promoters, including cytokinins, are rare. Extracted tRNA fractions from yeasts *Kluyveromyces lactis* (formerly *Saccharomyces lactis*) and *Saccharomyces cerevisiae* contained cytokinins (Armstrong et al., 1969). Immunological methods and chromatography detected cytokinins, and zeatin in particular, in the brewer’s yeast extract (Staden, 1974; Jameson & Morris, 1989). Furthermore, *Saccharomyces cerevisiae* and *Schizosaccharomyces pombe* were capable of tRNA-independent synthesis of cytokinins (Laten & Zahareas-Doktor, 1985). Cytokinin production by filamentous fungi has been reported previously (Murphy et al., 1997, López-Carbonell, Moret & Nadal, 1998, Morrison et al., 2015, Chanclud & Morel, 2016). The concentration of cytokinins increased in plant tissues infected with fungal plant pathogens (López-Carbonell, Moret & Nadal, 1998; Angra-Sharma & Sharma, 2000). However, a few studies suggested that fungal infection was not necessarily associated with increased production of cytokinins in plants, but rather resulted from fungal metabolic reactions (LeJohn & Stevenson, 1973; Barker & Tagu, 2000; Chanclud et al., 2016). The diversity of fungi that produce cytokinins is largely unknown. A few studies suggest that this ability might be more common among biotrophic than saprotrophic fungi (Murphy et al., 1997; Chanclud & Morel, 2016). Yeasts are a taxonomically heterogenous group of fungi comprising members of both basidiomycetes and ascomycetes. They are frequently found in association with plants (Fonseca & Inácio, 2006) and have been demonstrated to produce plant hormones, like the aforementioned IAA (reviewed by Kemler et al., 2017). In the present study we hypothesized that the ability to produce zeatin in yeasts can be as widespread as the synthesis of IAA. Therefore, we studied a collection of 76 wild strains of 36 yeast species (23 genera) and tested it for the production of zeatin using HPLC-MS/MS. Here, we provide the first comprehensive report of yeasts producing cytokinin zeatin in culture.

## Materials and methods

### Yeast isolates

Yeast strains were obtained from the collection of the Department of Soil Biology of the Lomonosov Moscow State University (WDCM CCINFO number: 1173; catalog: https://depo.msu.ru/). Cultures were isolated in the period of 2008–2015 and stored in a biochemically inactive state (in 15% glycerol at −80 °C) before experiments. A total of 76 strains of 36 species isolated from plant material (phyllosphere, flowers, roots, exudates, mosses), algae, soil, sediment and insects were included in the study (Table S1). Information about location and climate is also provided in Table S1. All strains used in this study were isolated in previous surveys (for example Kachalkin et al., 2008; Kachalkin & Yurkov, 2012; Kachalkin, 2014). Routinely, isolation of yeasts is followed by an MSP-fingerprinting (mini- and microsatellite PCR with, depending on a species, primers M13 or GTG_5_) and sequencing of rDNA of representative strains. Thus, only strains showing different MSP-PCR profiles are selected for sequencing and deposited to the collection. All studied strains were identified using partial rDNA sequencing of LSU rRNA gene (D1/D2 domains) and the ITS region as described before (Glushakova et al., 2015; Glushakova et al., 2016). Nucleotide sequences of the studied strains deposited in the GenBank are given in Table S1.

### Cultivation

A 5-days old culture grown on solid Glucose-Peptone-Yeast extract medium was used for cultivation experiments. An inoculum of two loops was transferred into a 50 ml tube containing 10 ml of 0.5% Glucose—YNB (Yeast Nitrogen Base, Fluka) medium (Kurtzman & Fell, 1998) and incubated for 3 days at 20 °C shaking at Rotamax 120 (Heidolph, Schwabach, Germany) shaker at 100 rpm. Then, 100 μl of the grown culture was transferred into a 200 ml flask (Greiner, Kremsmünster, Austria) containing 50 ml of same medium and incubated for 10 days (for an accumulation curve) or 3 days (for screening) as described above. After cultivation, liquid medium was separated from yeast cells by centrifugation for 10 min at 11,500 g. Yeast biomass was weighed after drying to a constant weight in a solid state thermostat at 50 °C for 3 days.

### Zeatin extraction

To extract zeatin, the collected liquid medium was acidified with formic acid to pH 3 by adding 500 μl of one M formic acid to 50 ml of the medium. The method was developed to work with the water base culture medium. The Dowex 50W×8 cation exchange resin (Sigma, Tokyo, Japan) was used for purification and concentration. Therefore, one ml of the resin was conditioned by successive passing five ml of methanol and five ml of one M formic acid. The culture medium was passed through the cation exchange resin at the rate of one drop/s. The culture medium that passed through the resin was discarded and the resin was washed with five ml of one M formic acid. Zeatin was eluted with six ml of 0.35 M ammonium hydroxide. The eluate was neutralized with 220 μl of five M formic acid. The neutralized extract was applied to the concentrating cartridge С18 Cromabond 500 mg (Sorbtech, Norcross, GA, USA) that was pre-conditioned by successive passing five ml of methanol and five ml of deionized water at the rate of one drop/s. The concentrate that passed through the cartridge was discarded and the cartridge was rinsed with five ml of deionized water. Zeatin was eluted from the cartridge with six ml of methanol into a distillation flask (Dobrev & Kamínek, 2002; Hoyerová et al., 2006) and concentrated on a rotary evaporator (50 rpm) at 30 °C (Lu et al., 2010) to a final volume of ca. 0.5 ml. The resulting concentrate was transferred to a 1.5 ml chromatography vial, and 0.5 ml of acetonitrile was added to a single-neck round-bottom flask which was then placed into an ultrasonic bath cleaner for 1 min to wash the remaining zeatin from the walls of the flask. The acetonitrile that was used to wash the flask was also transferred to the vial. The washing step was repeated twice. The final volume in the vial was adjusted to 1.5 ml with acetonitrile.

**Figure 1:**
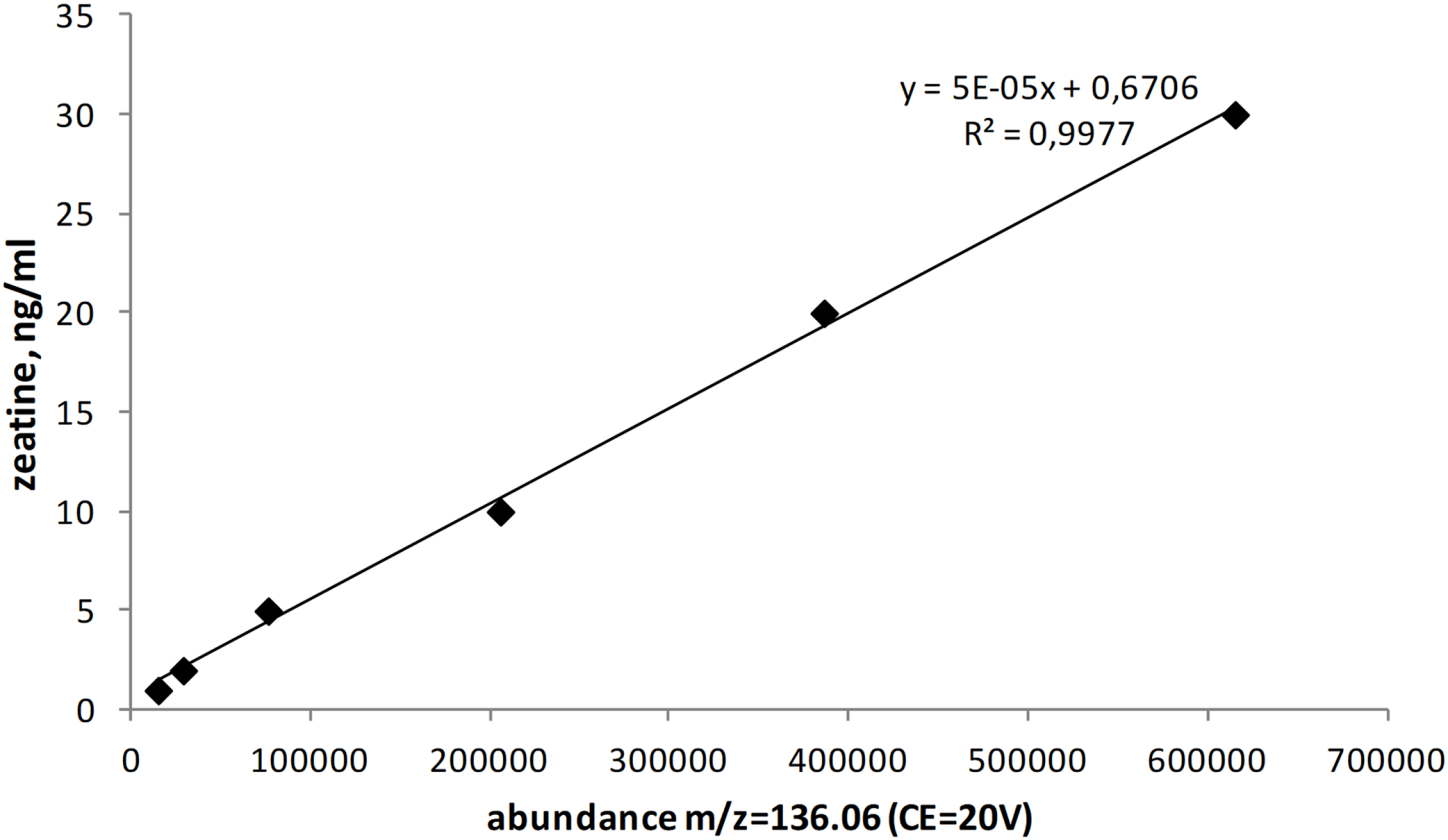
Calibration curve for zeatin.

The quantitation of zeatin was performed on a High-Performance Liquid Chromatography System Agilent 1200 series with a Quadrupole Time-of-Flight Mass Spectrometer 6520 Accurate-Mass Q-TOF LC/MS (Agilent Technologies, Santa Clara, CA, USA) (Novák et al., 2008; Pan & Wang, 2009) under following conditions, the ionization source: electrospray (+), CE = 20V, ion precursor: 220.12 m/z; daughter ions: 136.06 m/z (quantitative determination); confirming ion: 202.10 m/z; column: HPLC RP-8 3.5 μm 4.6×200 mm (Hewlett Packard, Palo Alto, CA, USA). The mobile phase was 10 mM formic acid and acetonitrile. The gradient table is shown below (Table 1). Retention time was 9.5 min and the volume of injected sample was 25 μl. The temperature of the thermostat was 30 °C. An analytical standard of a mixture of cis and trans-zeatin isomers (Acros) dissolved in acetonitrile was used as a reference. The calibration curve contained six levels (1, 2, 5, 10, 20 and 30 ng/ml). *R*^2^ = 0.998 (Fig. 1). Zeatin produced by yeasts was expressed as per gram of dry biomass.

**Table 1:** Chromatographic gradient.

Time, min	Acetonitrile, %	Flow rate, ml/min
0	20	0.5
5	50	0.5
12	50	0.5
12.5	100	0.5
15	100	0.5
15.5	20	0.5

### Statistical Analyses

Statistical differences were determined by pairwise comparisons of phylogenetic, geographic and ecological yeast groups with the nonparametric Mann–Whitney U-test and Wald-Wolfowitz runs test using STATISTICA 10 (StatSoft, Inc., Tulsa, OK, USA). Statistical significance was judged at the level of *p* < 0.05. Zero values were not taken into consideration

## Results

Zeatin was observed in 42 out of 76 strains (55%) included in the experiment (Table S1). Zeatin was successfully measured in 11 (35%) and in 31 (65%) of strains of ascomycetes and basidiomycetes, respectively. The average value of zeatin was 1,549.5 ng/g. Slightly higher values (*p* < 0.02, Wald-Wolfowitz test) were observed for ascomycetous than for basidiomycetous yeasts, that is 2,078.5 ng/g and 1,361.8 ng/g, respectively.

**Figure 2:**
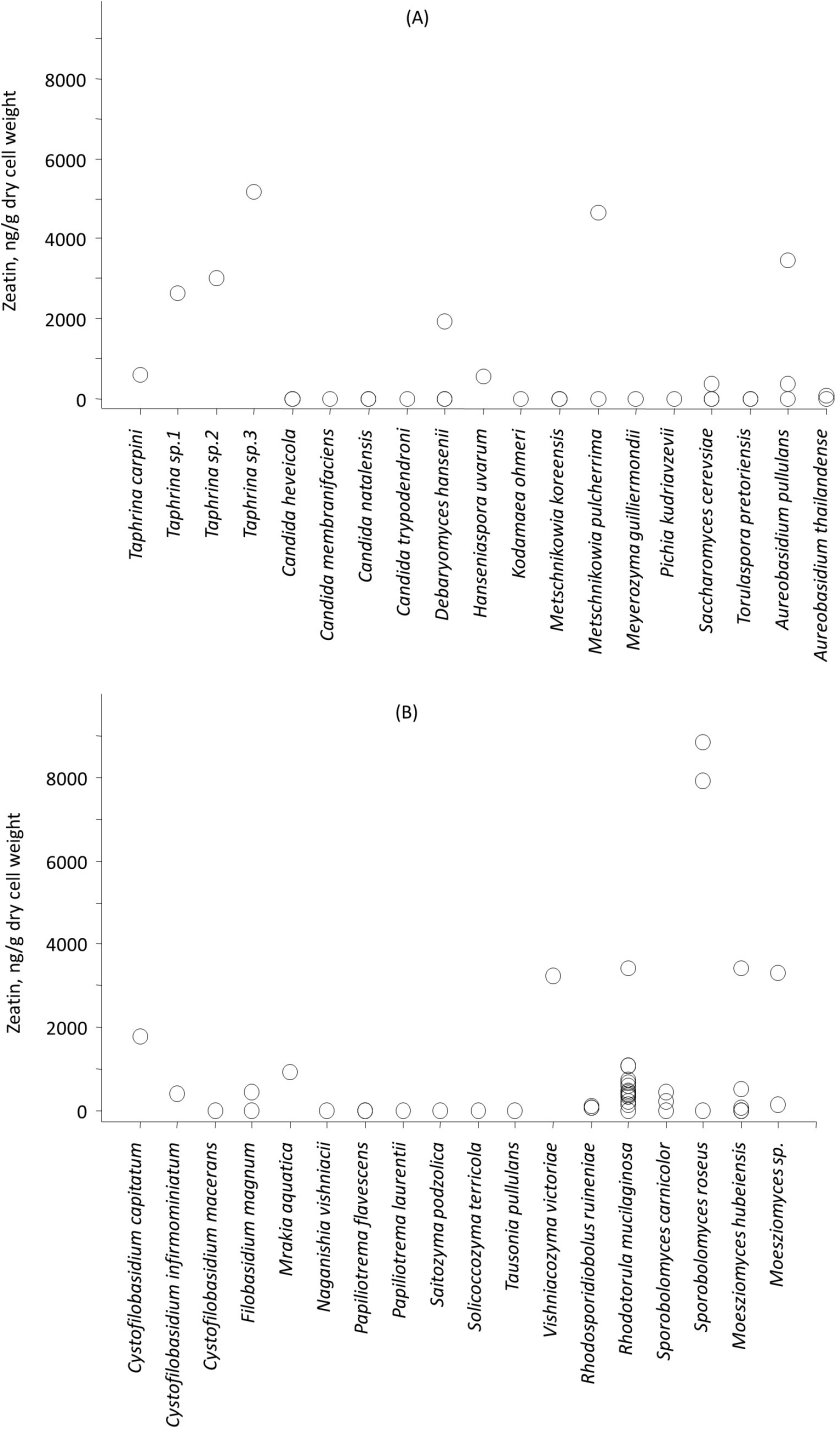
Average zeatin production (mean points) by yeast strains of phyla Ascomycota (A) and Basidiomycota (B).

Among studied strains highest zeatin values were observed for *Sporobolomyces roseus* strains KBP Y-5472 and KBP Y-5432, both isolated in the vicinity of the settlement of Dikson (Russian arctic, Kara sea) from plant surfaces. These yeasts produced 8,850.1 and 7,900.0 ng/g of zeatin, respectively. Originated from the same region yeast of the genus *Taphrina* (potential new species, strain KBP Y-5606) the highest production of the compound observed for ascomycetes, that is 5,166.4 ng/g of zeatin. Another ascomycete yeast, *Metschnikowia pulcherrima* strain KBP Y-6020 that was isolated from grapes in Dagestan (Russian subtropics) synthesized 4,633.0 ng/g of zeatin. Isolated from a tropical plant *Moesziomyces* (formerly *Pseudozyma*) *hubeiensis* strain KBP Y-5132 produced 3,423.8 ng/g of zeatin. Thus, strains characterized by higher values of zeatin were cultured from the phyllosphere. Among the soil-borne strains, the *Aureobasidium pullulans* KBP Y-5404 from a beech forest in Dagestan showed the highest value of 3,461.6 ng/g of zeatin. The ability to synthesize zeatin varied substantially among strains (Fig. 2). A widespread yeast species *Rhodotorula mucilaginosa* represented with 15 strains in this study showed the amount of zeatin ranging from 0 (below detection limit) to 3,409.9 ng/g. This observation goes in line with previous reports that showed that the production of another plant hormone IAA is strain-specific (Limtong & Koowadjanakul, 2012; Limtong et al., 2014; Jaiboon et al., 2016; Streletskii et al., 2016).

## Discussion

**Figure 3:**
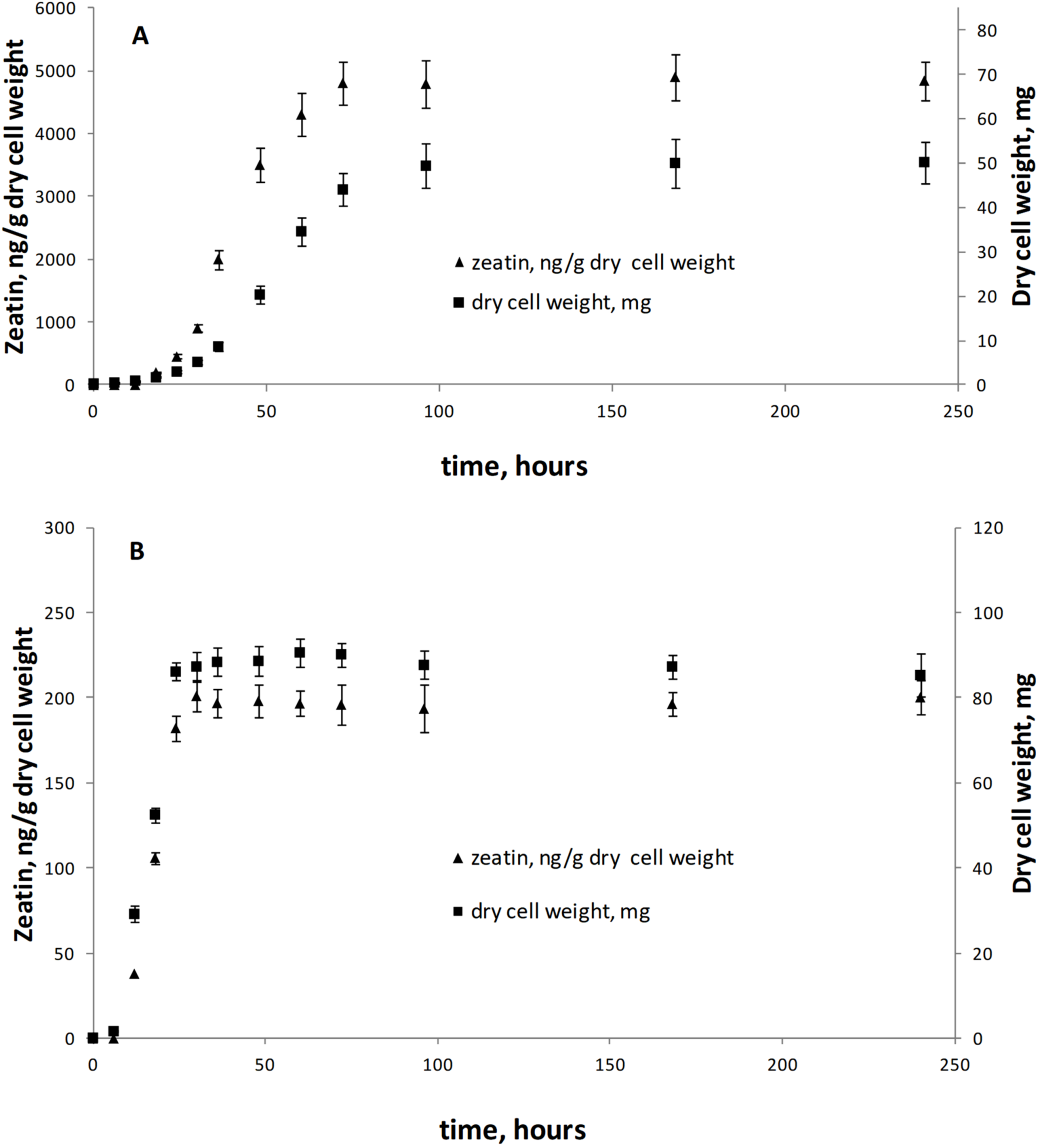
Zeatin production (triangle) and growth curves (square) of *Metschnikowia pulcherrima* KBP Y-6020 (A) and *Rhodotorula mucilaginosa* KBP Y-5419 (B). Bars are standard errors and middle points are the respective means.

**Figure 4:**
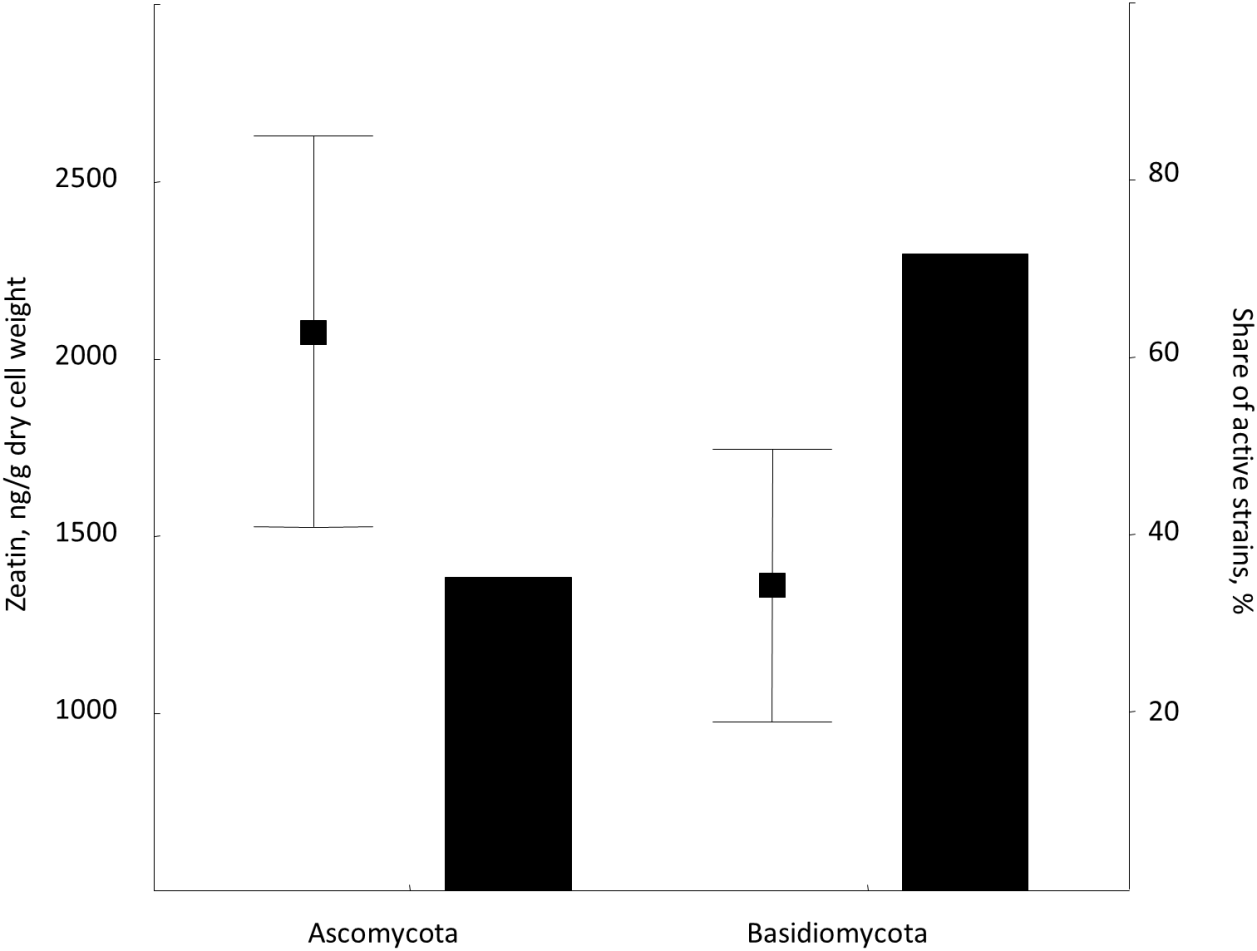
Average zeatin production (mean points with bars) and proportions of zeatin-producing strains (columns) in phyla Ascomycota and Basidiomycota. Bars are standard errors and middle points are the respective means.

This is the first broad study to evaluate the ability of yeasts to produce zeatin. A few previous studies investigated cytokinin production by yeasts. Specifically, 800 and 900 ng of N6 (Δ2-isopentenyl) adenosine per g of wet biomass were detected in an immunoassay for *Saccharomyces cerevisiae* and *Schizosaccharomyces pombe*, respectively (Laten & Zahareas-Doktor, 1985). These cytokinin concentrations are in the range observed in our study. In contrast, Jameson & Morris (1989) have detected only approximately 4.4 ng/g (20 pmol/g) of trans-zeatin in yeast extract of *Saccharomyces cerevisiae*, which is significantly lower than amounts found in our study. A few studies quantified cytokinins in filamentous fungi. Murphy et al. (1997) reported 27.4 ng/g (125 pmol/g) of zeatin riboside and 106 ng/g (486 pmol/g of wet weight) of isopentenyl adenosine in the mycelium of the ascomycete *Pyrenopeziza brassicae*, and a culture medium contained approximately 4.4 ng/g (125 pmol/g of wet biomass) of this compound. In *Thelephora terrestris*, cytokinins isopentenyl adenosine and zeatin riboside were found in the amount of ca. 3898 ng/g (17.8 pmol/mg of wet biomass) and 742 ng/g (3.39 pmol/mg), respectively (Kraigher et al., 1991). These results indicate that zeatin was previously found in rather small amounts, compared to results obtained in the present study (Table S1; Fig. 2). Nothing is known about dynamics of accumulation of zeatin in yeast culture. In filamentous fungi, trans-zeatin and trans-zeatin riboside were accumulated in the exponential growth phase, and isopentenyl adenine in the stationary phase (Van Staden & Nicholson, 1989). Similarly to filamentous fungi, we observed synthesis of zeatin by the ascomycetes *Metschnikowia pulcherrima* and basidiomycetes *Rodotorula mucilaginosa* in the exponential growth phase (Fig. 3). This observation differs, however, from results obtained for bacteria, which accumulate phytohormones linearly, that is the amount continuously increases both in the logarithmic and in the stationary phases of growth (Hussain & Hasnain, 2009; Kudoyarova et al., 2014). Although our study was not aimed to provide a solid analysis of zeatin production in yeasts from different taxonomical groups, habitats or climates, several observed trends are worth to mention. Most strains showed low production of zeatin but 10 strains (13% of all cultures) were able to synthesize substantial amounts of zeatin exceeding three ng/g (Table S1; Fig. 2). Some valid difference (Wald–Wolfowitz test) between members of ascomycetes and basidiomycetes (Fig. 4) were observed, but for subphyla no statistically supported difference was found.

Among ascomycetes, cultures of dimorphic plant pathogens from *Taphrinomycotina* of the genus *Taphrina* were characterized by an increased production of zeatin synthesis (Fig. 2). *Taphrina cerasi* and *Taphrina deformans* were reported to synthesize cytokinins in the amount of five and eight μg/g of dry weight in the equivalent of kinetin, respectively (Johnston, 1973). Taking into consideration that these results refer to the total amount of cytokinins, we assume that the amount of zeatin could be in the same range as values observed in our study. The relevance of cytokinin production for *Taphrina* is unknown, but either the role in parasitic or saprobic phase is feasible. It should be noted that *Taphrina* strains in this study were isolated from healthy plants and in the climatic zone (subarctic) that is not typical for these fungi due to the lack of common hosts. The loss of the sexual parasitic stage by some of these yeasts and their transition to solely saprobic lifestyle has been hypothesized, in particular for recently isolated from Antarctic cultures (Inácio et al., 2004, Selbmann et al., 2014). A positive effect of phyllosphere-inhabiting *Taphrina* on plants in cold climates cannot be excluded. Conversely, a series of symptoms observed in plants and characterized by abnormal tissue growth (e.g. extremely densely branched shoots know as witch’s broom) can be caused by over-expression of cytokinins in plants or result from a *Taphrina* (e.g. *Taphrina betulina* on birch) infection. In contrast to *Taphrina*, another prominent group of dimorphic plant parasites, members of *Ustilaginomycotina* (e.g. smuts, Begerow et al., 2014) from *Moesziomyces* genus, did not synthesize substantial amounts of zeatin (Fig. 2), although production of cytokinins by *Mycosarcoma maydis* has been previously reported (Mills & Van Staden, 1978). The concentration of cytokinins increases in the tissues of infected plants, and this response correlated with the virulence of the *Mycosarcoma maydis* (Bruce, Saville & Emery, 2011, Morrison et al., 2015). The contribution of cytokinins synthesized by the fungus to the phytohormone pool in host tissues has also been confirmed in experiments with mutants deficient in cytokinin production (Morrison, Emery & Saville, 2017). *Moesziomyces antarcticus* (formerly *Pseudozyma antarctica*) is a commercial producer of enzymes and glycolipid surfactants (Saika et al., 2014). Our study shows that *Moesziomyces* yeasts, specifically *Moesziomyces hubeiensis*, was able to synthesize zeatin.

*Taphrina* and yeasts formerly classified in the genus *Pseudozyma* (asexual Ustilaginomycetes) are both biotrophic plant parasites. However, they show a remarkable difference in distribution and growth in the host. Species of the genus *Taphrina* often infect plants of the northern hemisphere (Rodrigues & Fonseca, 2003) and develop intercellularly in plant tissues (Bassi, Conti & Barbieri, 1984). Members of Ustilaginomycotina (including *Moesziomyces* and *Pseudozyma* from this study) are more abundant in the tropical region (e.g. Begerow et al., 2014; Khunnamwong, Jindamorakot & Limtong, 2018) and characterized by intracellular propagation in host tissues (Begerow et al., 2014). The present study aimed to measure zeatin in yeasts sampled from different taxonomic groups and habitats. The selection of strains does not allow us to draw a well-supported conclusion regarding a possible role of zeatin synthesis in the growth of plant pathogens. Nevertheless, our results showed that the two groups of dimorphic pathogens contain strains, which are capable to synthesize high amount zeatin exceeding 3,000 ng/g. It has been also demonstrated that asexual states of *Ustilaginomycotina* intensively produce auxin in culture (Streletskii et al., 2016). Whether or not synthesis of plant hormones are relevant for the development of plant pathogenic fungi *in plantae* or for survival outside the host requires additional testing with more species and strains included in future studies.

**Figure 5:**
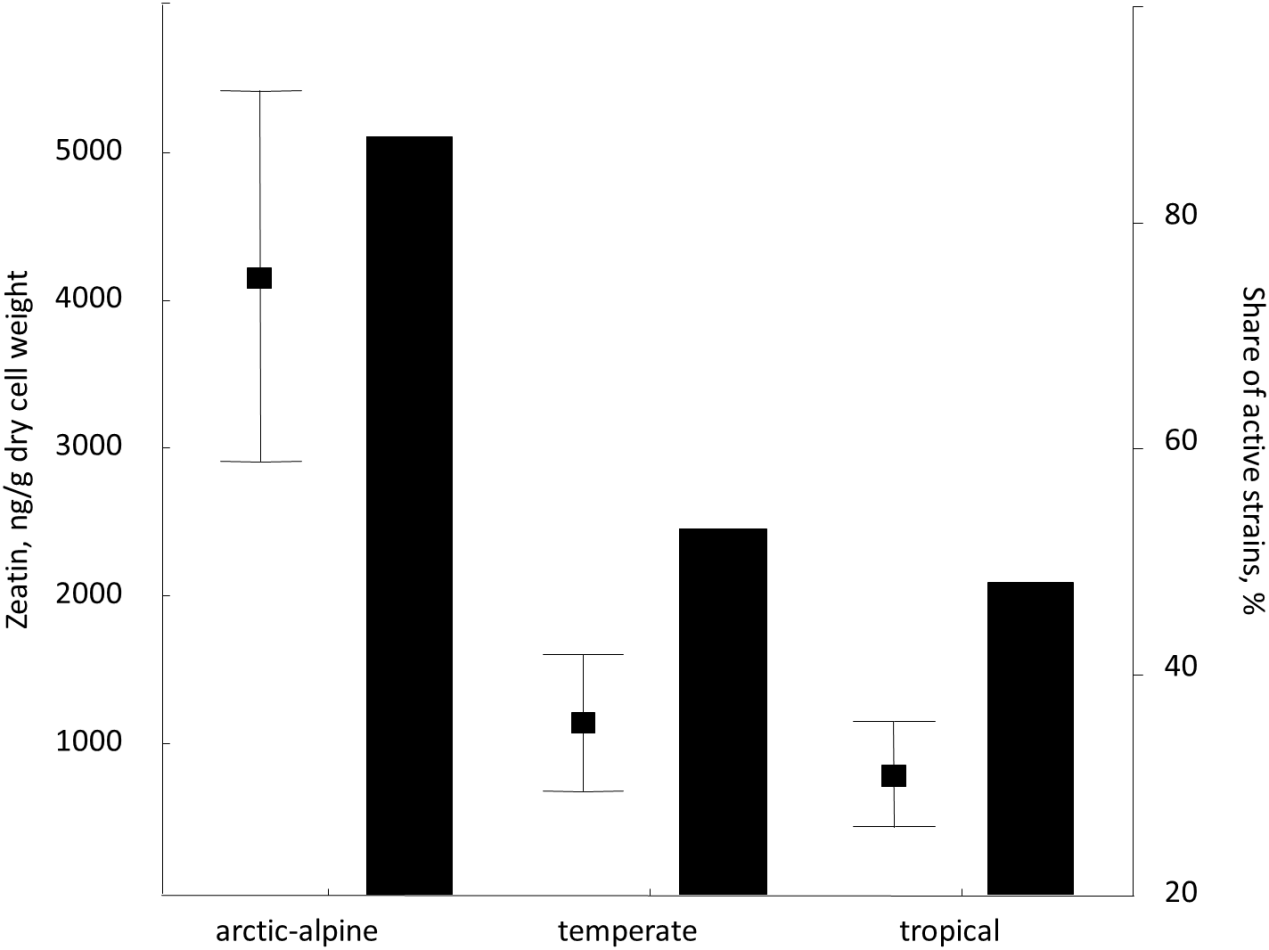
Average zeatin production (mean points with bars) and proportions of zeatin-producing strains (columns) among phylloplane strains grouped according to the region of isolation (Supplementary Table S1). Bars are standard errors and middle points are the respective means.

Despite rather limited number of strains included in the study, we attempted to analyze the distribution of zeatin-producing yeasts in relation to their origin, namely locality and substrate. Therefore, we compared zeatin values in strains from arctic-alpine, temperate and tropical zones (Whittaker, 1975). Among studied yeasts from vascular plants (most representative group), isolates from the arctic-alpine zone were more likely to synthesize zeatin (detected experimentally) and usually in higher values (Fig. 5). Specifically, zeatin was detected in seven out of eight strains (87.5%) from the arctic-alpine zone, while only nine out of the 17 strains (52.9%) from the temperate zone and only 12 out of the 25 strains (48.0%) from the tropical zone produced this phytohormone. This is in contrast with previous observations of auxin (IAA) synthesis by yeasts that showed higher values of this compound produced by yeasts from the tropical climate (Limtong et al., 2014; Streletskii et al., 2016). Zeatin values for plant-related isolates from arctic-alpine zone demonstrated statistically support differences with temperate (*p* < 0.05, U-test) and tropical (*p* < 0.02, U-test) zones. These results are partly influenced by high zeatin values measured in *Taphrina* spp. and *Sporobolomyces roseus* strains exclusively isolated from Russian arctic (Table S1). The trend is less obvious when isolates of the widespread species *Rhodotorula mucilaginosa* are considered.

Despite the rather heterogeneous origin of selected strains, comparison of cultures from soil- and plant-related substrates was possible. Yeasts isolated from vascular plants tended to exhibit different rates in zeatin production in the laboratory experiment compared with soil-borne strains. Specifically, zeatin was detected in 28 out of 50 strains (56.0%) from plant-related substrates, while only four active strains (44.4%) were found among isolates from soil. Many of yeasts that are commonly found on plant surfaces produce red-colored pigmentation (Fonseca & Inácio, 2006). Irrespective of their origin, pigmented yeasts were more likely to synthesize zeatin in our study. Carotenoid pigmentation in ascomycetous yeasts is restricted to members of *Taphrinomycotina*, namely genera *Protomyces* and *Taphrina* strains (Valadon, 1964), produced a substantial amount of zeatin in our study (Table S1; Fig. 2). Another pigmented ascomycetes yeasts from subphylum *Pezizomycotina* of the genus *Aureobasidium*, the most typical among epiphytes (Fonseca & Inácio, 2006), demonstrated the ability of zeatin production (Table S1; Fig. 2). Zeatin was detected in seven out of ten strains (70.0%) of pigmented ascomycetes, while only in four out of 22 strains (18.2%) of not pigmented ascomycetes, including *Metschnikowia pulcherrima*, produced this phytohormone in this study (Table S1; Fig. 2). Among the pigmented basidiomycetes, 23 out of 27 strains (85.2%) produced zeatin, whereas eight out of 18 non-pigmented yeasts (44.4%) yielded this compound in a detectable amount. Although pigmentation is a frequent trait among phylloplane yeasts, some prominent plant dwellers are not colored, including *Metschnikowia pulcherrima*, *D. hansenii* and members of genus *Pseudozyma* (Table S1). Interestingly, high zeatin production values were measured in cultures of these yeasts.

Yeasts are common inhabitants of plant surfaces, tissues and fluids (reviewed in Fonseca & Inácio, 2006; Kemler et al., 2017; Limtong & Nasanit, 2017). They can be beneficial for plants as antagonists of pathogens and act as plant growth promoters (reviewed in Kemler et al., 2017). This study demonstrated that, in addition to auxin, yeasts are able to produce a plant hormone zeatin and this ability is rather common among yeasts. Among factors considered to explain the observation of zeatin in yeasts, an association with a plant was a common feature joining ecologically and taxonomically distinct organisms, that is biotrophic plant pathogens and pigmented and non-pigmented phylloplane yeasts. Although the number of studied strains does not allow us to draw strong conclusions regarding the influence of phylloplane yeasts on plant development, our results suggest that plant-yeast interactions should be studied in more details in the future. In our opinion, it is also important to investigate the production of plant hormones by yeasts on and in plants.

Upon planning this experiment, we perceived that there is little overlap between yeast species inhabiting plants in cold and warm climates (reviewed by Kemler et al., 2017; Limtong & Nasanit, 2017; Yurkov, 2017). Originally unintended, plant-related yeasts from cold regions were found to be active producers of zeatin in our experiment. It has been suggested that a short vegetative period requires fast plant growth and microbial recruiting by plants (such as pigmented yeasts) could be important for ecological adaptation of plants to life in cold environments (Buzzini, Turchetti & Yurkov, 2018). We suppose that one of the mechanisms promoting plant growth in high latitudes could be cytokinins from external sources such as phyllosphere yeasts of genera *Rhodotorula*, *Sporoblomyces*, *Vishniacozym*a (e.g. Fonseca & Inácio, 2006; Kemler et al., 2017). In agreement with this expectation, we found that a few members of these genera were able to synthesize substantial amounts of zeatin. Additionally, we found that ascomycetes *Debaryomyces hansenii* and *Metschnikowia pulcherrima* could be an important source of zeatin in plant phyllosphere.

Yeasts were reported to produce plant hormones, either auxin or zeatin, or both. Still, the relevance of these compounds for yeast ecology is little known. Although many phylloplane yeasts possess this interesting ability, we think that it is premature to consider it as a distinctive ecological trait. It has been reported that both auxin (e.g. Limtong et al., 2014; Streletskii et al., 2016) and zeatin (this study) synthesis in culture varies between species and strains, and may depend on culture conditions (Scarcella et al., 2017). At present, our observations are more relevant for a targeted isolation of yeasts producing plant growth promoters than for plant and yeast ecologists. We believe that future studies in plants will bring better understanding of plant-yeast interactions involving plant hormones.

## Supplemental Information

10.7717/peerj.6474/supp-1 Supplemental Information 1 Raw data (screening study and zeatine accumulation).

10.7717/peerj.6474/supp-2 Supplemental Information 2 Zeatin production and characteristics of the tested yeast strains.

## Additional Information and Declarations

### Data Availability

The following information was supplied regarding data availability: The raw data are available in a Supplemental File.
