# Supplementary material for: Yeasts producing zeatin
Source: PeerJ. 2019 Feb 20;7:e6474. doi: 10.7717/peerj.6474 (PMC6387580; doi:10.7717/peerj.6474)
Supplement: Supplemental Information 2 [file peerj-07-6474-s002.docx]

**Yeasts producing zeatin**

Authors:

Rostislav A. Streletskii^1^; Aleksey V. Kachalkin^1^; Anna M. Glushakova^1^; Andrey M. Yurkov^2^; Vladimir V. Demin^1^

Affiliations:

^1^ Faculty of Soil Science, Lomonosov Moscow State University, Leninskie Gory 1, 119991 Moscow, Russia

^2^ Leibniz Institute DSMZ-German Collection of Microorganisms and Cell Cultures, Inhoffenstrasse 7B, 38124 Braunschweig, Germany

Corresponding authors

Correspondence to Rostislav A. Streletskii

Table 1. Zeatin production and characteristics of the tested yeast strains (±STD, n=2)

| strain | Organism | Sequence_ID | Isolation source | place | Collection Date | zeatin,ng/g dry weight |
| --- | --- | --- | --- | --- | --- | --- |
| **Phylum *Ascomycota*** | | | | | | |
| **Subphylum *Saccharomycotina*** | | | | | | |
| KBP Y-4891 | *Candida heveicola* | MF927603 | *Hevea brasiliensis* latex | Vietnam, Phu Cuong | 09/11/2014 | *n.d. |
| KBP Y-4892 | *Candida heveicola* | MF927604 | *Hevea brasiliensis* latex | Vietnam, Phu Cuong | 09/11/2014 | n.d. |
| KBP Y-5008 | *Candida membranifaciens* | MF927605 | grapes | Russia, Dagestan | 2011 | n.d. |
| KBP 11-29 | *Candida natalensis* | MF927606 | tropical flowers | Vietnam, Cat Tien | 25/11/2015 | n.d. |
| KBP 2-8 | *Candida natalensis* | MF927607 | Honeycomb, *Apis mellifera* | Vietnam, Cat Tien | 25/11/2015 | n.d. |
| KBP Y-5475 | *Candida trypodendroni* | MF927608 | termites, *Nasutitermes sp.* | Vietnam, Cat Tien | 9/11/2015 | n.d. |
| KBP Y-4569 | *Debaryomyces hansenii* | MF927612 | leaves of *Chamaedaphne calyculata* | Russia, Moscow region | 15/12/2008 | n.d. |
| KBP Y-4869 | *Debaryomyces hansenii* | MF927613 | Hevea brasiliensis latex | Vietnam, Phu Cuong | 9/11/2014 | n.d. |
| KBP Y-4781 | *Debaryomyces hansenii* | MF927614 | filamentous algae | Russia, Moscow region | 19/05/2011 | 1946,0±352,5 |
| KBP Y-5021 | *Hanseniaspora uvarum* | MF927617 | grapes | Russia, Dagestan | 2011 | 553,6±74,9 |
| KBP 13-7 | *Kodamaea ohmeri* | MF927618 | guts of Diplopoda indet. | Vietnam, Cat Tien | 25/11/2015 | n.d. |
| KBP 33-19 | *Metschnikowia koreensis* | MF927619 | tropical flowers | Vietnam, Cat Tien | 25/11/2015 | n.d. |
| KBP 15-20 | *Metschnikowia koreensis* | MF927620 | tropical flowers | Vietnam, Cat Tien | 25/11/2015 | n.d. |
| KBP Y-5623 | *Metschnikowia pulcherrima* | MF927621 | grapes | Russia, Dagestan | 1/09/2011 | n.d. |
| KBP Y-6020 | *Metschnikowia pulcherrima* | MF927622 | grape leaves | Russia, Dagestan | 30/09/2012 | 4633,0±633,9 |
| KBP Y-5005 | *Meyerozyma guilliermondii* | MF927628 | grapes | Russia, Dagestan | 2011 | n.d. |
| KBP Y-5020 | *Pichia kudriavzevii* | MF927629 | grapes | Russia, Dagestan | 2011 | n.d. |
| KBP Y-4511 | *Saccharomyces cerevisiae* | MF927655 | roots of *Carex limosa* | Russia, Moscow region | 15/02/2009 | n.d. |
| KBP Y-5009 | *Saccharomyces cerevisiae* | MF927656 | grapes | Russia, Dagestan | 2011 | 378,9±60,4 |
| KBP Y-4748 | *Saccharomyces cerevisiae* | MF927657 | *Sphagnum sp.* | Russia, Moscow region | 15/05/2008 | n.d. |
| KBP Y-5478 | *Torulaspora pretoriensis* | MF927671 | litter | Vietnam, Cat Tien | 9/11/2015 | n.d. |
| KBP 5-5 | *Torulaspora pretoriensis* | MF927672 | guts of *Diplopoda* indet. | Vietnam, Cat Tien | 25/11/2015 | n.d. |
| **Subphylum *Pezizomycotina*** | | | | | | |
| KBP Y-5464 | *Aureobasidium pullulans* | MF927598 | *Dryas punctata* | Russia, Dikson | 1/08/2012 | n.d. |
| KBP Y-5396 | *Aureobasidium pullulans* | MF927599 | surfase of bison mummy | Russia, Chukotka Autonomous Area | 1/06/2012 | 383,1±48,7 |
| KBP Y-5404 | *Aureobasidium pullulans* | MF927600 | soil under beech forest | Russia, Dagestan | 29/06/2012 | 3461,6±480,9 |
| KBP 11-17 | *Aureobasidium thailandense* | MF927601 | tropical flowers | Vietnam, Cat Tien | 25/11/2015 | n.d. |
| KBP 11 -18 | *Aureobasidium thailandense* | MF927602 | tropical flowers | Vietnam, Cat Tien | 25/11/2015 | 84,8±13,9 |
| **Subphylum *Taphrinomycotina*** | | | | | | |
| KBP Y-5582 | *Taphrina carpini* | MF927666 | plants | Russia, Dikson | 1/08/2012 | 591,2±86,0 |
| KBP Y-4841 | *Taphrina sp.* | MF927667 | *Sphagnum sp.* | Russia, Taymyr | 2010 | 2640,5±454,9 |
| KBP Y-5581 | *Taphrina sp.* | MF927668 | *Dryas punctata* | Russia, Dikson | 1/08/2012 | 3024,2±531,5 |
| KBP Y-5606 | *Taphrina sp.* | MF927669 | *Dryas punctata* | Russia, Dikson | 1/08/2012 | 5166,4±829,4 |
| **Phylum *Basidiomycota*** | | | | | | |
| **Subphylum *Agaricomycotina*** | | | | | | |
| KBP Y-4518 | *Cystofilobasidium capitatum* | MF927609 | *Ceratophyllum sp.* | Russia, Moscow | 19/05/2011 | 1791,4±313,1 |
| KBP Y-4998 | *Cystofilobasidium infirmominiatum* | MF927610 | soil | Russia, Syktyvkar | 2011 | 404,3±66,2 |
| KBP Y-4993 | *Cystofilobasidium macerans* | MF927611 | *Potamogeton natans* | Russia, Moscow | 19/05/2011 | n.d. |
| KBP Y-4534 | *Filobasidium magnum* | MF927615 | lake silt | Russia, Moscow region | 15/06/2011 | 451,9±76,4 |
| KBP Y-4668 | *Filobasidium magnum* | MF927616 | *Ledum palustre* | Russia, Khanty-Mansi Autonomous Area – Yugra | 1/10/2008 | n.d. |
| KBP Y-5620 | *Mrakia aquatica* | MF927623 | algae from soil surface | Russia, Dikson | 1/08/2012 | 920,3±155,6 |
| KBP Y-5000 | *Naganishia vishniacii* | MF927624 | *Elodea canadensis* | Russia, Moscow | 19/05/2011 | n.d. |
| KBP 33-23 | *Papiliotrema flavescens* | MF927625 | tropical flowers | Vietnam, Cat Tien | 25/11/2015 | n.d. |
| KBP 33-6 | *Papiliotrema flavescens* | MF927626 | tropical flowers | Vietnam, Cat Tien | 25/11/2015 | n.d. |
| KBP 1-26 | *Papiliotrema laurentii* | MF927627 | tropical flowers | Vietnam, Cat Tien | 25/11/2015 | n.d. |
| KBP Y-4614 | *Saitozyma podzolica* | MF927658 | soil | Russia, Kaluga region | 17/09/2014 | n.d. |
| KBP Y-4724 | *Solicoccozyma terricola* | MF927659 | soil | Russia, Taymyr | 2010 | n.d. |
| KBP Y-4999 | *Tausonia pullulans* | MF927670 | soil | Russia, Syktyvkar | 2011 | n.d. |
| KBP Y-5562 | *Vishniacozyma victoriae* | MF927673 | *Dryas punctata* | Russia, Dikson | 1/08/2012 | 3219,6±562,6 |
| **Subphylum *Ustilaginomycotina*** | | | | | | |
| KBP Y-4938 | *Pseudozyma sp.* | MF927630 | plants | Vietnam, Cat Tien | 1/11/2014 | 131,5±23,5 |
| KBP Y-5446 | *Pseudozyma hubeiensis* | MF927631 | flowers of *Dipterocarpus sp.* | Vietnam, Cat Tien | 21/11/2015 | n.d. |
| KBP 33-8 | *Pseudozyma hubeiensis* | MF927632 | tropical flowers | Vietnam, Cat Tien | 25/11/2015 | n.d. |
| KBP 35-7 | *Pseudozyma hubeiensis* | MF927633 | plants | Vietnam, Cat Tien | 25/11/2015 | 60,7±8,6 |
| KBP Y-5116 | *Pseudozyma hubeiensis* | MF927634 | plants | Sri Lanka | 6/06/2015 | 505,8±88,5 |
| KBP Y-5132 | *Pseudozyma hubeiensis* | MF927635 | plants | Sri Lanka | 6/06/2015 | 3423,8±461,8 |
| KBP Y-5492 | *Pseudozyma sp.* | MF927636 | tropical flowers | Vietnam, Cat Tien | 1/08/2012 | 3316,2±486,2 |
| **Subphylum Pucciniomycotina** | | | | | | |
| KBP 11-15 | *Rhodosporidiobolus ruineniae* | MF927637 | tropical flowers | Vietnam, Cat Tien | 21/11/2015 | 65,1±10,5 |
| KBP Y-5448 | *Rhodosporidiobolus ruineniae* | MF927638 | tropical flowers | Vietnam, Cat Tien | 1/08/2012 | 68,4±11,3 |
| KBP 33-15 | *Rhodosporidiobolus ruineniae* | MF927639 | tropical flowers | Vietnam, Cat Tien | 21/11/2015 | 95,2±11,9 |
| KBP Y-5300 | *Rhodotorula mucilaginosa* | MF927640 | soil | Russia, Kaluga region | 17/09/2014 | n.d. |
| KBP Y-4763 | *Rhodotorula mucilaginosa* | MF927641 | *Sphagnum magellanicum* | Russia, Khanty-Mansi Autonomous Area – Yugra | 1/10/2008 | 154,0±26,4 |
| KBP Y-5419 | *Rhodotorula mucilaginosa* | MF927642 | *Lycopodium sp.* | Russia, Dikson | 1/08/2012 | 207,2±39,1 |
| KBP Y-5693 | *Rhodotorula mucilaginosa* | MF927643 | *Stipa sp.* | Mongolia: Ulaanbaatar | 17/09/2009 | 325,6±45,4 |
| KBP Y-5692 | *Rhodotorula mucilaginosa* | MF927644 | *Geum rivale* | Russia: Moscow region | 2/06/2008 | 371,5±68,9 |
| KBP Y-5881 | *Rhodotorula mucilaginosa* | MF927645 | *Deschampsia alpina* | Norway: Svalbard | 1/3/2011 | 387,1±70,0 |
| KBP 473-2 | *Rhodotorula mucilaginosa* | MF927646 | *Sphagnum angustifolium* | Russia: Tver' region | 1/06/2008 | 411,1±61,8 |
| KBP Y-5689 | *Rhodotorula mucilaginosa* | MF927647 | soil | Norway: Svalbard | 1/3/2011 | 428,2±66,7 |
| KBP Y-4736 | *Rhodotorula mucilaginosa* | MF927648 | *Sphagnum angustifolium* | Russia: Tver' region | 1/06/2008 | 468,8±90,8 |
| KBP Y-4528 | *Rhodotorula mucilaginosa* | MF927649 | soil | Morocco | 10/02/2011 | 613,4±103,1 |
| KBP 965k | *Rhodotorula mucilaginosa* | MF927650 | *Carex limosa* | Russia: Moscow region | 15/02/2009 | 661,7±120,8 |
| KBP Y-4669 | *Rhodotorula mucilaginosa* | MF927651 | *Sphagnum sp.* | Russia: Tver' region | 1/06/2008 | 725,2±114,0 |
| KBP Y-4737 | *Rhodotorula mucilaginosa* | MF927652 | *Chamaedaphne calyculata* | Russia: Moscow region | 15/08/2008 | 1093,9±193,8 |
| KBP Y-5285 | *Rhodotorula mucilaginosa* | MF927653 | plants | Malaysia: Borneo | 1/05/2011 | 1092,0±206,3 |
| KBP Y-5007 | *Rhodotorula mucilaginosa* | MF927654 | soil | Russia: Komi Republic | 2011 | 3409,9±524,7 |
| KBP 16-29 | *Sporobolomyces carnicolor* | MF927660 | flowers of *Dipterocarpus sp.* | Vietnam: Cat Tien | 25/11/2015 | n.d. |
| KBP 11-13 | *Sporobolomyces carnicolor* | MF927661 | tropical flowers | Vietnam: Cat Tien | 25/11/2015 | 226,5±37,8 |
| KBP Y-5444 | *Sporobolomyces carnicolor* | MF927662 | tropical flowers | Vietnam: Cat Tien | 12/11/2015 | 435,5±69,1 |
| KBP Y-5408 | *Sporobolomyces roseus* | MF927663 | marine algae | Russia: Karelia | 13/06/2011 | n.d. |
| KBP Y-5432 | *Sporobolomyces roseus* | MF927664 | *Lycopodium sp.* | Russia: Dikson | 9/11/2015 | 7900,0±1117,2 |
| KBP Y-5472 | *Sporobolomyces roseus* | MF927665 | plants | Russia: Dikson | 14/11/2015 | 8850,1±1299,2 |

* not detected
